# When Classic Signs Deceive: A Widespread Papulosquamous Eruption in Skin of Colour

**DOI:** 10.3390/dermatopathology12030021

**Published:** 2025-07-21

**Authors:** Ji Fung Yong, Claudine Howard-James, Stephen Crowther, Anne-Marie Tobin, Caitriona Hackett

**Affiliations:** 1 Department of Dermatology, Tallaght University Hospital, D24 NR0A Tallaght, Ireland; 2 Department of Histopathology, Tallaght University Hospital, D24 NR0A Tallaght, Ireland; 3 School of Medicine, Trinity College Dublin, D02 PN40 Dublin, Ireland

**Keywords:** autoinflammatory disorders, dermatopathology, lichen planus, medical dermatology

## Abstract

A 29-year-old gentleman of African descent presented to the emergency department with a three month history of a rash affecting the trunk, upper limbs, and thighs. The patient was unsure of any triggers and denied any preceding illness, new medications, illicit drug use, or recent vaccinations. On examination, there was a widespread papulosquamous eruption characterised by scaly, hyperpigmented papules and plaques involving the trunk, upper arms, and upper thighs. A definitive diagnosis was established through a diagnostic skin biopsy of a fresh lesion.

## 1. Case Presentation

A 29-year-old gentleman of African descent presented to the emergency department with a three-month history of a rash affecting the trunk, upper limbs, and thighs. The rash initially began in the groin area and subsequently spread to the aforementioned areas. It was raised, associated with intense itching, and occasionally accompanied by a stinging sensation. The patient was unsure of any triggers and denied any preceding illness, new medications, illicit drug use, recent vaccinations or recent travel. He had no underlying medical conditions but had been treated for a *Chlamydia trachomatis* infection six months prior to the onset of the rash. Repeat sexually transmitted disease screening four months earlier was negative. Systematic review was unremarkable.

**Figure 1:**
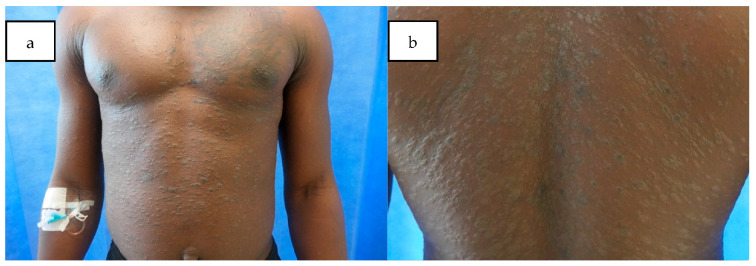
Papulosquamous eruption with scaly, hyperpigmented papules and plaques affecting the upper arms, anterior and posterior trunk (**a**); a close-up view of the rash on the back (**b**).

Full skin examination revealed a widespread papulosquamous eruption characterised by scaly, hyperpigmented papules and plaques affecting the trunk, upper arms, and upper thighs (Figure 1a,b). There was no involvement of palmoplantar surfacers, nails, ocular or oral mucosal surfaces. Routine blood investigations were within normal limits. Blood serology for HIV, hepatitis B and C, and syphilis, was negative. Skin scrapings for fungal studies were negative.

**Figure 2:**
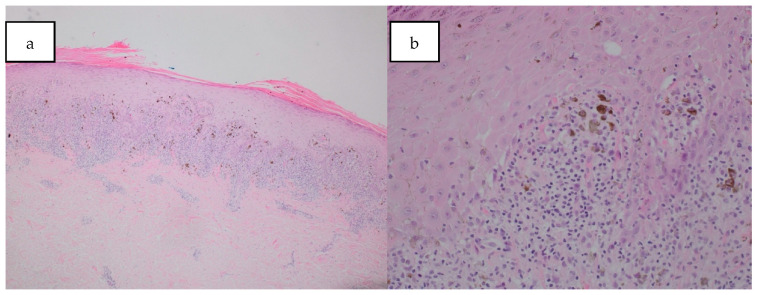
(**a**) Histology slide showed epidermal hyperkeratosis, hypergranulosis, and irregular acanthosis, with a band-like lymphocytic infiltrate at the dermo-epidermal junction. Haematoxylin and Eosin stain a. Magnification ×4; (**b**) histology slide shows Civatte bodies at the interface. Haematoxyin and Eosin stain. Magnification ×20.

A skin biopsy from a plaque on the abdomen revealed epidermal hyperkeratosis, hypergranulosis, and irregular acanthosis. There was moderate dermal chronic inflammation, characterised by a band-like lymphocytic infiltrate at the dermo-epidermal junction, with the presence of apoptotic keratinocytes (Civatte bodies) (Figure 2a,b). Eosinophils were absent. Gram, Giemsa and periodic-acid–Schiff stains were negative.

What is the diagnosis?

- Cutaneous lichen planus (LP);
- Plaque psoriasis;
- Atopic dermatitis;
- Pityriasis rosea;
- Tinea Corporis.

Diagnosis:

Cutaneous lichen planus (LP).

While the clinical presentation of this papulosquamous eruption could suggest all the other options, further history and investigations ruled out these differential diagnoses.

Plaque psoriasis is incorrect. Plaque psoriasis is an immune-mediated, genetic disease that affects the skin, joints, or both. There is usually a family history of plaque psoriasis. The hallmark of psoriasis on histology includes epidermal acanthosis, hyperkeratosis and parakeratosis [1]. However, there is an absence of a lichenoid band infiltrate at the dermo-epidermal junction in psoriasis.

Similarly, atopic dermatitis is incorrect. This patient has no personal or family history of atopy. Furthermore, histology usually demonstrates spongiosis and dermal oedema in the acute phase; acanthosis, hyperkeratosis and parakeratosis of the epidermis in subacute lesions; and varying thickness of the granular layer secondary to rubbing and scratching [2].

Pityriasis rosea is a common papulosquamous eruption that occurs in healthy adolescents and young adults. It typically presents with a “Herald patch” that appears before the generalised rash. Diagnosing pityriasis rosea may be challenging, and histological features usually include spongiotic dermatitis, along with elongation of the rete ridges and enlargement of the dermal papillae, all of which were absent in this case. This condition is usually self-limiting [3].

Tinea corporis is incorrect as skin scrapings for fungal elements were negative. This was further confirmed by a negative periodic acid–Schiff stain.

## 2. Discussion

Lichen planus (LP) is a chronic inflammatory skin condition which affects approximately 0.1 to 1.27% of the general population [4]. While the autoimmune mechanism of LP is well-established, the underlying aetiology remains poorly understood. It is postulated that activated T cells accumulates in the dermis and are responsible for the apoptosis of the basal epidermal cells. In addition to immune dysregulation, other factors such as infections (hepatitis B and C, varicella zoster and human herpesviruses 6 and 7), immunisation, genetic associations and environmental risk factors (e.g., emotional distress and colouring substances) have been proposed as potential aetiologies for LP [5].

LP typically presents as a papulosquamous eruption with flat-topped, violaceous papular lesions, often described using the “6 P’s”: purple, polygonal, planar, pruritic, papules, and plaques. It commonly affects the extremities, with Wickham striae visible under dermoscopy. This patient initially attended the GP, but a definitive diagnosis was not made. Instead, he was prescribed a trial of a short course of topical betamethasone valerate, which unfortunately failed to improve the rash. Due resistance of the rash and associated itch, he subsequently presented to the casualty department for further evaluation. In skin of colour, LP may not exhibit the typical “6 P’s” descriptions but may instead appear darker than the background skin colour, as seen in our patient, which can pose a challenge in reaching a clinical diagnosis.

Given the widespread distribution, our initial differential diagnoses included atopic dermatitis, guttate psoriasis, pityriasis rosea, tinea corporis, eczematous or lichenoid drug reactions and secondary syphilis. The definitive diagnosis was confirmed through histopathological findings in a skin biopsy taken from a fresh, untreated lesion. A thorough history taking was also crucial in reaching the diagnosis in this patient, especially since the classic signs may not be seen, to exclude systemic causes such as drug reaction or underlying skin conditions. Generalised involvement of LP, as seen in our patient, is rare and has typically observed to adopt a Blaschkoid, intertriginous or dermatomal configuration [5]. Generalised LP has been reported in association with medications such as antimalarials, methyldopa, gold, or tumour necrosis factor-alpha blockers, as well as vaccinations^4^. Other potential triggers include viral infections [6] or skin injury (Koebnerisation), although none of these were relevant in this case.

Secondary syphilis is a great mimicker and was considered initially due to the history of chlamydia infection in our patient. However, secondary syphilis typically presents with annular cutaneous lesions consisting of peripheral erythematous papules with central hyperpigmentation in Black patients [7]. Additionally, secondary syphilis was ruled out by blood tests and histological findings.

The goal of treatment is to alleviate the symptoms and shorten the interval between symptom onset and remission. First-line treatment includes topical corticosteroids, topical calcineurin inhibitors and intralesional triamcinolone injections for localised disease. Systemic corticosteroids, acitretin and oral ciclosporin can be considered for more extensive disease. Second-line management involves narrowband UVB, with or without acitretin [8]. Additionally, Janus kinase (JAK) inhibitors have been successful in the treatment of all forms of LP. A literature review has shown rapid alleviation of LP symptoms, superior efficacy in numerous cases of LP that were refractory to other treatments, and excellent tolerability without serious side effects [9]. It has also been proposed that the IL-23/IL-17 pathway plays an important role in the pathogenesis of LP, indicating potential roles for anti-IL-23/IL-17 therapies in managing patients with LP [9]. Given the extensive disease, our patient is currently receiving narrow-band UVB phototherapy in conjunction with low-dose acitretin. He has experienced significant relief from itching, flattening of the lesions, and continues to show improvement.

This case highlights the challenges of diagnosing common inflammatory dermatoses such as LP in patients with skin of colour, where the classic features may not be immediately recognisable. A thorough history taking, complemented by appropriate diagnostic tools such as blood screening for infections and histopathology, is crucial to achieving an accurate diagnosis.

## Data Availability

No new data were created or analysed in this study. Data sharing is not applicable to this article.
